# Understanding Dupuytren's Disease Using Systems Biology: A Move Away from Reductionism

**DOI:** 10.3389/fphys.2012.00316

**Published:** 2012-08-09

**Authors:** Samrina Rehman, Philip J. R. Day, Ardeshir Bayat, Hans V. Westerhoff

**Affiliations:** ^1^Manchester Centre for Integrative Systems Biology, University of ManchesterManchester, UK; ^2^Quantitative Molecular Medicine Research, Centre for Integrated Genomic Medical Research, University of ManchesterManchester, UK; ^3^Plastic and Reconstructive Surgery Research, Manchester Institute of Biotechnology, University of ManchesterManchester, UK; ^4^Doctoral Training Centre Integrative Systems Biology from Molecule to Life, The Manchester Centre for Integrative Systems Biology, University of ManchesterManchester, UK; ^5^Netherlands Institute for Systems Biology, VU University AmsterdamAmsterdam, Netherlands; ^6^Synthetic Systems Biology, Swammerdam Institute for Life Sciences, University of AmsterdamAmsterdam, Netherlands

## Introduction

Dupuytren's disease (DD) resides within the poorly understood, yet important category of superficial quasi-neoplastic proliferative fibromatosis (McFarlane et al., 1990). This nodular palmar fibromatosis often causes permanent flexion contracture of the metacarpophalangeal (MCP) and proximal interphalangeal joints (PIPJ) of the digits (Rayan, 2007) leading to loss of function (Horner and Bralliar, 1971; Schroter, 1971; Tubiana, 1971; Rayan, 2007). DD may invade locally within the palmar aponeurosis of the hand (sparingly supplied with blood vessels). DD does not disseminate to other tissues (Seemayer et al., 1980), but, rather behaves as a benign neoplastic disorder: progressive and irreversible with a high rate of recurrence after surgical excision (current gold standard treatment for DD; Bayat and McGrouther, 2006). The increasing severity and aggressive recurrence may lead to amputation of the affected digit (Shaw et al., 2007).

Phenotypically, DD is described by its two distinct fibrotic elements: the nodule and cord. Nodules are highly vascularized, soft-tissue masses containing mostly myofibroblasts, while the cords are relatively avascular with a thickened collagen-rich structure (Rayan, 2007; Rehman et al., 2008, 2011). Different schools of thought exist regarding the definition of DD progression; they debate whether the nodule develops into cord or the two fibrotic elements occur independently from a separate precursor cell. Processes associated with DD pathogenesis include cytokines, growth factors, adhesion molecules, and extracellular matrix components (Rehman et al., 2008; Shih et al., 2009). Free radicals and localized ischemia may trigger the proliferation of DD tissue (Murrell et al., 1987). Histology has confirmed the presence of collagens, myofibroblast, and myoglobin proteins in DD, but at widely varying abundances. Pathophysiology of DD is also thought to arise either from a defect in a wound repair process or from an abnormal response to wounding (Shih and Bayat, 2010). Some of these hypotheses are non-molecular or may associate the disease with effects, rather than with a single cause. No animal model exists for the study of DD fibromatosis, yet investigations in animal studies of possibly related diseases (Akai et al., 1997) may be informative (Tart and Dahners, 1989; Kandel et al., 1991; Akai et al., 1997; Hildebrand et al., 2004). Single gene changes have correlated poorly with DD, and DD is nowadays viewed as a complex disease. We propose that DD may be a network disease, such that a systems biology approach may help its understanding.

## How Systems Biology Can Assist

Abnormal development such as in DD and cancer can influence activities within the surrounding tissues. Hence an association of these diseases with normal tissue repair processes should be expected, compromising the distinction between the genes causing the development of such a disease and the genes involved in the homeostatic response. Response to perturbations may be regulated at different levels [Daran-Lapujade et al., 2007 and processes are linked extensively, if not through metabolic or gene expression networks, then through RNAs (sense, anti-sense, or micro; Hendrickson et al., 2009)], or through dynamic ultrastructure (Westerhoff et al., 1990, 2009a; van Driel et al., 2003). To understand disease one therefore needs to look at the operation and integration of several simultaneous processes, as a function of time. Since the sum of positive (regulation) and negative (homeostasis) effects tend to decide the outcome, the approach needs to be precise experimentally and quantitative in the analysis, and relate to molecular biology and functional genomics as well as physiology (Westerhoff and Palsson, 2004).

Systems approaches have invalidated the concept of one “rate limiting” step determining metabolic pathways (Groen et al., 1982), gene expression circuits (Koster et al., 1988; Snoep et al., 2002; Stuger et al., 2002), and signal transduction (Hornberg et al., 2005a,b). A similar scenario is expected for DD, and can be tested by exploiting methodologies developed on simpler systems (Westerhoff, 2008; Westerhoff et al., 2009a,b). The impact of network theory (Barabási and Albert, 1999) on understanding has been substantial especially so in gene networks (Alon, 2007), metabolic networks (Schuster et al., 2002; Reed and Palsson, 2004; Westerhoff, 2008), in plant systems biology (Marshall et al., 2007), and even in food webs (Getz et al., 2003). Biological networks adapt and change temporally (Reijenga et al., 2005), are hierarchical in terms of space, time and organization (Westerhoff et al., 2009a), and have been optimized through evolution for multiple properties that evade our understanding (Schuetz et al., 2007; Westerhoff, 2008; Westerhoff et al., 2009b).

New network theories are targeted more toward comprehending biological systems functionally (Molenaar et al., 2009). The Flux-Balance-Analysis objective function of maximum growth yield for instance (Edwards et al., 2002) is irrelevant for the human erythrocyte and muscle cell (Westerhoff et al., 2009b). The objective of oxygen carrying capacity plus binding capacity in the lungs and delivery capacity in the tissues, and maximum performance independent of oxygen makes more sense. It remains to be seen whether in DD the flux pattern adjusts to a new criterion of optimality, such as in some cases of tumorigenesis (Rodríguez-Enríquez et al., 2009).

If DD was triggered by a single genetic factor, then it should encounter so many diverse processes during its etiology that it will still be co-determined by the many factors regulating those processes. If the networks governing differentiation of normal fibroblasts of the palm of the hand are perturbed so as to cause differentiation into tissue that is muscle-like in terms of expressing alpha smooth-muscle actin and being contractile without the usual controllers of contraction and relaxation, then various sets of genetic perturbations could lead to DD. Here DD may be much like cancer (Hornberg et al., 2006). The dilemma is that although we now avail of an unprecedented set of methodologies for the identification and analysis of molecules in living cells, seeing more molecules may not help (Lazebnik, 2002). Visualizing the connections between them may heighten our understanding of DD.

## The Way Forward: A Coalition between Systems Biology and Molecular Analyses

### Anatomical model hooked to *in silico* model

Absence of animal models for DD can be offset to some extent by *in silico* models using integrated systems approaches. A virtual hand (Soh et al., 2009) may help elucidate functional outcomes/behaviors of the proliferation of diseased cells. Here to it should be useful to map expression patterns onto such an anatomical model and attach this to *in silico* models of metabolism and gene expression (Westerhoff, 2001). Such an approach could overcome the ethical issues in animal research studies (Westerhoff et al., 2008).

### Imaging

Perhaps the biggest growth area in imaging technologies (Megason and Fraser, 2007) is fluorescence imaging, adapted for *in vivo* analysis. Label-free methods such as Raman microspectrometry are also showing promise (Ellis and Goodacre, 2006). The former will allow researchers to assess how components of intracellular signaling pathways interact in real time (e.g., Maeder et al., 2007; Chen et al., 2008). Computed tomography (CT), magnetic resonance imaging (MRI), positron emission tomography (PET), confocal microscopy, cell imaging, and Raman microspectroscopy already provide a platform for potentiating a knowledge base relationship between the clinician, patient, and scientist. Integration of the observation platform with dynamic-spatial models (Soh et al., 2009) has not yet been achieved. Systems biology would help evaluate the metabolic state of the various cells in the nodules and the cords of DD as a function of contractile activity and assess the de- and re-differentiation of fibroblasts and myofibroblasts.

### Establish an optimum cell system to model DD

Several factors need to be taken into account when building a cellular model for DD (Rehman et al., 2012). Notably, differing numbers of cell passages have been reported in the studies investigating the Dupuytren tissue, and changes in the proliferative potential of the fibroblast unstated. DD fibroblasts may possess a higher potential for matrix and collagen production through passages than control fascia cells because the DD nodules and cords result from an uncontrolled proliferative cellular state. Differences in collagen, fibronectin proteins, matrix expression proteins, and even proteoglycans could be affected by cell passage because it is thought that at earlier passages all cells would mostly be proliferating while at later passages they would tend to become senescent (Benvenuti et al., 2002) or rather quiescent.

Careful consideration should be given to factors that affect the reproducibility of data or produce instrument drift including sample preparation, instrument contamination, and data processing. In addition to the obvious factors such as patient age, gender, disease grade, and recurrence, the time and order of sample analysis could provide significant sources of variability, potentially obscuring the biological variation which we seek to characterize.

### Discovery of pathway biomarkers through stress-induced stimuli in DD networks

Once the cell system is determined, high throughput studies can characterize disease using healthy cells to enable identification of pathways for the construction of disease-specific networks. A metabolomics approach would facilitate identification of involved metabolites, as well as the pathways in which they occur. Subsequently the integration of transcript and metabolite profiling data will determine congruence between the levels of certain metabolites, gene transcripts, and their protein product(s) to identify metabolic pathways, signaling pathways, and key networks (connections and intercellular dynamics) that may attribute to formation of DD. Next, the investigation of the cellular network response to stress-induced stimuli (e.g., response to xenobiotics) will assist the mechanistic and kinetic modeling of complete pathways. Key parameters (e.g., initial concentrations of enzymes and metabolites, enzyme kinetic properties such as *K*_m_, *k*_cat_, and *K*_i_), and variables (e.g., concentrations of metabolites and metabolic fluxes) of the DD and control dynamical system may then be assigned.

### Text mining tools, and SBML models for systems biology information extraction from DD and connective tissue disorders

In pharma, systems biology is increasingly adopted, as is the use of next generation sequencing tools, e.g., RNA Seq, enhancing further the impact microarrays have had on transcript wide screening in the last decade to investigate disease. More than >45,000 hits in PubMed using the term “systems biology” and >54,000 hits for “DNA microarray analysis” were retrieved in early 2012. In 6 months (July, 2010 to January, 2011) 6000+ papers with the term “microarray” in title or abstract were added to the database. However only ∼2100 publications on DD have appeared since the original publication by Guillaume Dupuytren in 1831.

Systems biology also tries to integrate all relevant literature data and pre-existing knowledge with new experimental data in its attempts to understand network functioning. To this aim, systematic text mining of the literature is important for extraction of experimentally validated biological entities related to DD, e.g., concentrations of metabolites, enzymes, genes, or other related disease. The rationale to develop novel DD and connective tissue-related tools, standard languages (SBML) and models may help to depict accurate relationships in bio-networks by using natural language processing approaches. Emerging SBML and various databases ranging from proteome–proteome interaction databases (Pagel et al., 2005), human metabolome database (HMDB; (Wishart et al., 2009), gene ontology (GO; (Ashburner et al., 2000) already facilitate bridging or integrating some of the existing data about DD into a knowledgebase.

### The clinic and its limitations

It should be recognized that increasing sample replications is not always possible, particularly for clinical samples where patients are under treatment and any new diagnostic test is a secondary goal. Therefore, there needs to be greater emphasis on more robust experimental design. For example, to avoid sample bias, adequate matching of patients with disease to those who are healthy, in terms of gender, age, BMI, and ethnicity, as well as other extrinsic factors such as metabolic rates, pharmaceutical use and diet. Multidisciplinary team are required. Transnational funding across three to four experts groups ranging from clinicians, biologists, instrumentalists, and systems biologists may allow critical mass to be established.

## Conclusion

Approximately 181 years have elapsed since the first DD literature reference. The research has so far implicated a differentiation of fibroblasts into myofibroblasts and highlighted multiple mRNAs of which the expression level differs between diseased and healthy tissues. Extracellular matrix enzymes are involved in the disease. High throughput and candidate-gene association studies have suggested multiple biomarkers for the disease, but these would require complex and invasive sampling. Thus in the absence of radically new findings, the present mainstream research paradigm is unlikely to lead to a full understanding of the disease. What is known about DD suggests it is a multifactorial network disease requiring targeting of a few complete sets of pathways. A part way house is needed whereby a systems biology lead is adopted and molecular data generated to test and colonize potential networks. The use of time and changes to microenvironment should be promoted. Single cell analyses too will help to assess the impact of cell location and cell–cell contact. Availability of different disease states and severities will help to define perturbations and chronograph network change and disease progression. This will also assist therapeutic target selection. A consortium effort to understand DD should be initiated and the parameters then correlated across the various levels of systemic description. This should enable a collective investigation of pathological mechanisms in DD and related fibrotic connective tissue disorders.

## References

[B1] Akai M. Shirasaki Y. Tateishi T. (1997). Electrical stimulation on joint contracture: an experiment in rat model with direct current. Arch. Phys. Med. Rehabil. 78, 405–409 10.1016/S0003-9993(97)90233-1 9111461

[B2] Alon U. (2007). Network motifs: theory and experimental approaches. Nat. Rev. Genet. 8, 450–461 10.1038/nrg2102 17510665

[B3] Ashburner M. Ball C. A. Blake J. A. Botstein D. Butler H. Cherry J. M. Davis A. P. Dolinski K. Dwight S. S. Eppig J. T. Harris M. A. Hill D. P. Issel-Tarver L. Kasarskis A. Lewis S. Matese J. C. Richardson J. E. Ringwald M. Rubin G. M. Sherlock G. (2000). Gene Ontology: tool for the unification of biology. Nat. Genet. 25, 25–29 10.1038/75556 10802651 PMC3037419

[B4] Barabási A.-L. Albert R. (1999). Emergence of scaling in random networks. Science 286, 509–512 10.1126/science.286.5439.509 10521342

[B5] Bayat A. McGrouther D. A. (2006). Management of Dupuytren's disease – clear advice for an elusive condition. Ann. R. Coll. Surg. Engl. 88, 3–8 10.1308/147870806X95203 16460628 PMC1963648

[B6] Benvenuti S. Cramer R. Bruce J. Waterfield M. D. Jat P. S. (2002). Identification of novel candidates for replicative senescence by functional proteomics. Oncogene 21, 4403–4413 10.1038/sj.onc.1205525 12080471

[B7] Chen Y.-C. M. Kappel C. Beaudouin J. Eils R. Spector D. L. (2008). Live cell dynamics of promyelocytic leukemia nuclear bodies upon entry into and exit from mitosis. Mol. Biol. Cell 19, 3147–3162 10.1091/mbc.E07-08-0735 18480407 PMC2441680

[B8] Daran-Lapujade P. Rossell S. Van Gulik M. W. Luttik M. A. H. De Groot M. J. L. Slijper M. Heck A. J. R. Daran J. De Winde J. H. Westerhoff H. V. Pronk J. T. Bakker B. M. (2007). The fluxes through glycolytic enzymes in *Saccharomyces cerevisiae* are predominantly regulated at posttranscriptional levels. Proc. Natl. Acad. Sci. U.S.A. 104, 15753–15758 10.1073/pnas.0707476104 17898166 PMC2000426

[B9] Edwards J. S. Covert M. Palsson B. (2002). Metabolic modelling of microbes: the flux-balance approach. Environ. Microbiol. 4, 133–140 10.1046/j.1462-2920.2002.00282.x 12000313

[B10] Ellis D. I. Goodacre R. (2006). Metabolic fingerprinting in disease diagnosis: biomedical applications of infrared and Raman spectroscopy. Analyst 131, 875–885 10.1039/b602376m 17028718

[B11] Getz W. M. Westerhoff H. V. Hofmeyr J.-H. S. Snoep J. L. (2003). Control analysis of trophic chains. Ecol. Modell. 168, 153–171 10.1016/S0304-3800(03)00208-4

[B12] Groen A. K. Wanders R. J. Westerhoff H. V. Vandermeer R. Tager J. M. (1982). Quantification of the contribution of various steps to the control of mitochondrial respiration. J. Biol. Chem. 257, 2754–2757 7061448

[B13] Hendrickson D. G. Hogan D. J. McCullough H. L. Myers J. W. Herschlag D. Ferrell J. E. Brown P. O. (2009). Concordant regulation of translation and mRNA abundance for hundreds of targets of a human microRNA. PLoS Biol. 7, e1000238 10.1371/journal.pbio.1000238 19901979 PMC2766070

[B14] Hildebrand K. Sutherland C. Zhang M. (2004). Rabbit knee model of post-traumatic joint contractures: the long-term natural history of motion loss and myofibroblasts. J. Orthop. Res. 22, 313–320 10.1016/j.orthres.2003.08.012 15013090

[B15] Hornberg J. J. Binder B. Bruggeman F. J. Schoeberl B. Heinrich R. Westerhoff H. V. (2005a). Control of MAPK signalling: from complexity to what really matters. Oncogene 24, 5533–5542 10.1038/sj.onc.1208817 16007170

[B16] Hornberg J. J. Bruggeman F. J. Binder B. Geest C. R. De Vaate A. J. M. B. Lankelma J. Heinrich R. Westerhoff H. V. (2005b). Principles behind the multifarious control of signal transduction. FEBS J. 272, 244–258 10.1111/j.1432-1033.2004.04404.x 15634347

[B17] Hornberg J. J. Bruggeman F. J. Westerhoff H. V. Lankelma J. (2006). Cancer: a systems biology disease. BioSystems 83, 81–90 10.1016/j.biosystems.2005.05.014 16426740

[B18] Horner R. L. Bralliar F. (1971). Dupuytren's contracture. Analysis of 100 consecutive surgical cases. Rocky Mt. Med. J. 68, 49–52 5097775

[B19] Kandel J. Bossy-Wetzel E. Radvanyi F. Klagsbrun M. Folkman J. Hanahan D. (1991). Neovascularization is associated with a switch to the export of bFGF in the multistep development of fibrosarcoma. Cell 66, 1095–1104 10.1016/0092-8674(91)90033-U 1717155

[B20] Koster J. G. Destrée O. H. J. Westerhoff H. V. (1988). Kinetics of histone gene expression during early development of *Xenopus laevis*. J. Theor. Biol. 135, 139–167 10.1016/S0022-5193(88)80071-7 3267765

[B21] Lazebnik Y. (2002). Can a biologist fix a radio? Or, what I learned while studying apoptosis. Cancer Cell 2, 179–182 10.1016/S1535-6108(02)00133-2 12242150

[B22] Maeder C. I. Hink M. A. Kinkhabwala A. Mayr R. Bastiaens P. I. H. Knop M. (2007). Spatial regulation of Fus3 MAP kinase activity through a reaction-diffusion mechanism in yeast pheromone signalling. Nat. Cell Biol. 9, 1319–1326 10.1038/ncb1652 17952059

[B23] Marshall A. Gollapudi S. De Silva J. Hodgman C. (2007). A systems biology approach to modelling tea (*Camellia sinensis*). BMC Syst. Biol. 1, P13 10.1186/1752-0509-1-S1-P13

[B24] McFarlane R. M. McGrouther D. A. Flint M. H. (eds). (1990). Dupuytren's Disease: Biology and Treatment. New York: Churchill Livingstone

[B25] Megason S. G. Fraser S. E. (2007). Imaging in systems biology. Cell 130, 784–795 10.1016/j.cell.2007.08.031 17803903

[B26] Molenaar D. Van Berlo R. De Ridder D. Teusink B. (2009). Shifts in growth strategies reflect tradeoffs in cellular economics. Mol. Syst. Biol. 5, 1–10 10.1038/msb.2009.82 PMC2795476 19888218

[B27] Murrell G. Francis M. Bromley L. (1987). Free radicals and Dupuytren's contracture. Br. Med. J. (Clin. Res. Ed.) 28, 1373–1375 10.1136/bmj.295.6610.1373 PMC1248535 2825907

[B28] Pagel P. Kovac S. Oesterheld M. Brauner B. Dunger-Kaltenbach I. Frishman G. Montrone C. Mark P. Stümpflen V. Mewes H.-W. Ruepp A. Frishman D. (2005). The MIPS mammalian protein-protein interaction database. Bioinformatics 21, 832–834 10.1093/bioinformatics/bti115 15531608

[B29] Rayan G. M. (2007). Dupuytren disease: anatomy, pathology, presentation, and treatment. J. Bone Joint Surg. Am. 89, 189–198 10.1302/0301-620X.89B2.18161 17256226

[B30] Reed J. Palsson B. (2004). Genome-scale in silico models of *E. coli* have multiple equivalent phenotypic states: assessment of correlated reaction subsets that comprise network states. Genome Res. 14, 1797–1805 10.1101/gr.2546004 15342562 PMC515326

[B31] Rehman S. Goodacre R. Day P. J. Bayat A. Westerhoff H. V. (2011). Dupuytren's: a systems biology disease. Arthritis Res. Ther. 13, 238 10.1186/ar3438 21943049 PMC3308066

[B32] Rehman S. Salway F. Stanley J. K. Ollier W. E. R. Day P. Bayat A. (2008). Molecular phenotypic descriptors of Dupuytren's disease defined using informatics analysis of the transcriptome. J. Hand. Surg. Am. 33, 359–372 10.1016/j.jhsa.2007.11.010 18343292

[B33] Rehman S. Xu Y. Dunn W. B. Day P. J. R. Westerhoff H. V. Goodacre R. Bayat A. (2012). Dupuytren's disease metabolite analyses reveals alterations following initial short-term fibroblast culturing. Mol. Biosyst. 8, 2274–2288 10.1039/c2mb25173f 22772395

[B34] Reijenga K. A. Bakker B. M. van der Weijden C. C. Westerhoff H. V. (2005). Training of yeast cell dynamics. FEBS J. 272, 1616–1624 10.1111/j.1742-4658.2005.04582.x 15794749

[B35] Rodríguez-Enríquez S. Marín-Hernández A. Gallardo-Pérez J. C. Carreño-Fuentes L. Moreno-Sánchez R. (2009). Targeting of cancer energy metabolism. Mol. Nutr. Food Res. 53, 29–48 10.1002/mnfr.200700470 19123180

[B36] Schroter G. (1971). The recognition of Dupuytren's contracture as an occupational disease. Beitr. Orthop. Traumatol. 18, 78–80 5575605

[B37] Schuetz R. Kuepfer L. Sauer U. (2007). Systematic evaluation of objective functions for predicting intracellular fluxes in *Escherichia coli*. Mol. Syst. Biol. 3, 1–15 10.1038/msb4100162 PMC1949037 17625511

[B38] Schuster S. Hilgetag C. Woods J. H. Fell D. A. (2002). Reaction routes in biochemical reaction systems: algebraic properties, validated calculation procedure and example from nucleotide metabolism. J. Math. Biol. 45, 153–181 10.1007/s002850200143 12181603

[B39] Seemayer T. Lagacé R. Schürch W. Thelmo W. (1980). The myofibroblast: biologic, pathologic, and theoretical considerations. Pathol. Annu. 15(Pt 1), 443–470 7443312

[B40] Shaw R. B. Chong A. K. S. Zhang A. Hentz V. R. Chang J. (2007). Dupuytren's disease: history, diagnosis, and treatment. Plast. Reconstr. Surg. 120, 44e–54e 10.1097/01.prs.0000278455.63546.03 17700106

[B41] Shih B. Bayat A. (2010). Scientific understanding and clinical management of Dupuytren disease. Nat. Rev. Rheumatol. 6, 715–726 10.1038/nrrheum.2010.180 21060335

[B42] Shih B. Wijeratne D. Armstrong D. J. Lindau T. Day P. Bayat A. (2009). Identification of biomarkers in Dupuytren's disease by comparative analysis of fibroblasts versus tissue biopsies in disease-specific phenotypes. J. Hand Surg. 34, 124–136 10.1016/j.jhsa.2008.09.017 19121738

[B43] Snoep J. L. van der Weijden C. C. Andersen H. W. Westerhoff H. V. Jensen P. R. (2002). DNA supercoiling in *Escherichia coli* is under tight and subtle homeostatic control, involving gene-expression and metabolic regulation of both topoisomerase I and DNA gyrase. Eur. J. Biochem. 269, 1662–1669 10.1046/j.1432-1327.2002.02803.x 11895436

[B44] Soh J. Turinsky A. L. Trinh Q. M. Chang J. Sabhaney A. Dong X. Gordon P. M. Janzen R. P. Hau D. Xia J. Wishart D. S. Sensen C. W. (2009). Spatiotemporal integration of molecular and anatomical data in virtual reality using semantic mapping. Int. J. Nanomedicine 4, 79–89 19421373 10.2147/ijn.s4375 PMC2720741

[B45] Stuger R. Woldringh C. L. Weijden V. D. Coen C. Vischer N. O. E. Bakker B. M. Spanning V. Rob J. M. Snoep J. L. Westerhoff H. V. (2002). DNA supercoiling by gyrase is linked to nucleoid compaction. Mol. Biol. Rep. 29, 79–82 10.1023/A:1020318705894 12241080

[B46] Tart R. P. Dahners L. E. (1989). Effects of electrical stimulation on joint contracture in a rat model. J. Orthop. Res. 7, 538–542 10.1002/jor.1100070411 2786955

[B47] Tubiana R. (1971). Dupuytren's disease. Present status of the treatment. Cah. Med. 12, 1305–1309 5157743

[B48] van Driel R. Fransz P. F. Verschure P. J. (2003). The eukaryotic genome: a system regulated at different hierarchical levels. J. Cell. Sci. 116, 4067–4075 10.1242/jcs.00779 12972500

[B49] Westerhoff H. V. (2001). The silicon cell, not dead but live! Metab. Eng. 3, 207–210 10.1006/mben.2001.0192 11461142

[B50] Westerhoff H. V. (2008). Signalling control strength. J. Theor. Biol. 252, 555–567 10.1016/j.jtbi.2007.11.035 18222488

[B51] Westerhoff H. V. Aon M. A. Vandam K. Cortassa S. Kahn D. van Workum M. (1990). Dynamic and hierarchical coupling. Biochim. Biophys. Acta 1018, 142–146 10.1016/0005-2728(90)90235-V

[B52] Westerhoff H. V. Kolodkin A. Conradie R. Wilkinson S. Bruggeman F. Krab K. Van Schuppen J. Hardin H. Bakker B. Moné M. Rybakova K. N. Eijken M. Van Leeuwen H. Snoep J. (2009a). Systems biology towards life in silico: mathematics of the control of living cells. J. Math. Biol. 58, 7–34 10.1007/s00285-008-0160-8 18278498

[B53] Westerhoff H. V. Winder C. Messiha H. Simeonidis E. Adamczyk M. Verma M. Bruggeman F. J. Dunn W. (2009b). Systems biology: the elements and principles of life. FEBS Lett. 583, 3882–3890 10.1016/j.febslet.2009.11.018 19913018

[B54] Westerhoff H. V. Mosekilde E. Noe C. R. Clemensen A. M. (2008). Integrating systems approaches into pharmaceutical sciences. Eur. J. Pharm. Sci. 35, 1–4 10.1016/j.ejps.2008.05.011 18602464

[B55] Westerhoff H. V. Palsson B. O. (2004). The evolution of molecular biology into systems biology. Nat. Biotechnol. 22, 1249–1252 10.1038/nbt1020 15470464

[B56] Wishart D. S. Knox C. Guo A. C. Eisner R. Young N. Gautam B. Hau D. D. Psychogios N. Dong E. Bouatra S. Mandal R. Sinelnikov I. Xia J. Jia L. Cruz J. A. Lim E. Sobsey C. A. Shrivastava S. Huang P. Liu P. Fang L. Peng J. Fradette R. Cheng D. Tzur D. Clements M. Lewis A. De Souza A. Zuniga A. Dawe M. Xiong Y. Clive D. Greiner R. Nazyrova A. Shaykhutdinov R. Li L. Vogel H. J. Forsythe I. (2009). HMDB: a knowledgebase for the human metabolome. Nucleic Acids Res. 37, D603–D610 10.1093/nar/gkn810 18953024 PMC2686599

